# Optimization of Fluorinated Ether-Based Quasi-Solid Electrolyte Systems for Lithium–Sulfur Batteries

**DOI:** 10.1021/acsaem.6c00080

**Published:** 2026-03-11

**Authors:** Ishani Senevirathna, Changlong Chen, Junquan Ou, Vignyatha Tatagari, Leon Shaw, Carlo U. Segre

**Affiliations:** † Department of Physics, 2455 Illinois Institute of Technology, Chicago, Illinois 60616, United States; ‡ Department of Mechanical, Materials, and Aerospace Engineering, 2455 Illinois Institute of Technology, Chicago, Illinois 60616, United States

**Keywords:** quasi-solid state, lithium−sulfur batteries, modified mixture design, Gaussian process regression, in situ polymerization

## Abstract

Although quasi-solid-state lithium–sulfur (Li–S) batteries show great promise for safe and high-energy storage systems, optimizing electrolyte formulations remains challenging due to the complex interplay of factors such as ion transport, stability, and sulfur utilization. In this study, seven quasi-solid electrolyte formulations were systematically investigated based on a ternary electrolyte component system of 1,3-dioxolane (DOL), 1*H*,1*H*,5*H*-octafluoropentyl 1,1,2,2-tetrafluoroethyl ether (OTE), and 1,2-dimethoxyethane (DME). The seven electrolyte formulations were designed based on a modified mixture design adapted from the design of experiments (DoE) principles. A Gaussian process regression (GPR) model was then used to statistically map the relationship between electrolyte composition and performance responses. Here, GPR is used as a data-driven approximation to capture composition–performance trends and guide electrolyte optimization within the ternary design space. The electrolytes were formed via *in situ* polymerization to ensure mechanical stability and maintain favorable interfacial contact with the electrodes. The model, trained on experimental data, identified an optimized composition (DOL:OTE:DME = 0.273:0.505:0.222) with improved predicted performance compared to the initial set. The optimized electrolyte delivered a high initial discharge capacity of 861 mAh g^–1^ at 0.3 C with only 9.2% capacity loss over 100 cycles showing markedly improved cycling stability compared to the baseline electrolyte. The statistical modeling provides a powerful framework for electrolyte development and offers valuable insights into the composition–performance relationships in multicomponent electrolyte systems.

## Graphical abstract

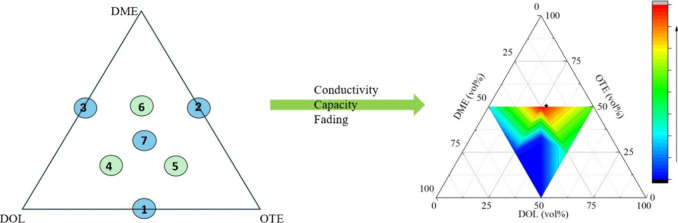

## 1 Introduction

Lithium–sulfur (Li–S) batteries are widely considered potential candidates for next-generation energy storage systems due to the natural abundance, low cost, and nontoxicity of sulfur, along with its high theoretical specific capacity of 1675 mAh g^–1^.^1,2^ Sulfur cathodes typically exhibit favorable electrochemical performance in ether-based electrolytes, owing to their good compatibility. Among the commonly used solvents, 1,2-dimethoxyethane (DME) offers low viscosity and a high dielectric constant, enabling enhanced ionic conductivity and more complete redox reactions at the sulfur cathode. 1,3-Dioxolane (DOL) contributes to improved cycling stability by limiting the solubility of lithium polysulfides (LiPSs) and promoting the formation of a more stable solid electrolyte interphase (SEI) on the lithium anode.^3,4^

Despite these advantages, the practical deployment of Li–S batteries remains hindered by the dissolution of intermediate LiPSs and the resulting polysulfide shuttle effect, which leads to capacity fading and low Coulombic efficiency (CE).^5^ To overcome these challenges, recent research has focused on optimizing the sulfur host materials^6^ and the electrolyte formulation.^7^ Recently, highly concentrated electrolytes (HCEs) have proven effective in suppressing dendrite growth and mitigating the polysulfide shuttle effect in Li–S batteries. These systems enable high-rate cycling of lithium metal with high CE, largely due to the strong coordination between lithium ions and solvents in the high-salt environment, which regulates Li_2_S_*n*_ solubility and reaction dynamics.^8,9^ However, the use of large quantities of lithium salt in HCEs results in drawbacks such as high cost, poor wettability, high viscosity, and low ionic conductivity.^10^ To address these limitations while retaining the advantages of HCEs, localized high-concentration electrolytes (LHCEs) have been developed by introducing an inert diluent, which preserves the solvation structure while reducing salt content. In ether-based electrolytes, solvents with high donor numbers improve salt dissolution; however, they simultaneously enhance polysulfide solubility, intensifying the shuttle effect. In contrast, fluorinated ethers with low donor numbers, low permittivity, and low viscosity serve as diluents in LHCEs, maintaining the lithium-ion coordination environment, suppressing polysulfide dissolution, and improving electrolyte wettability.^11^ Additionally, the choice of diluent influences SEI formation, which is crucial for enhancing cycling stability and battery lifespan. Zheng et al. demonstrated that an ether-based electrolyte, 1*H*,1*H*,5*H*-octafluoropentyl-1,1,2,2-tetrafluoroethyl ether (OTE), effectively suppressed polysulfide dissolution and dendrite growth in Li–S batteries, achieving a high CE of 99.2% and stable cycling performance.^12^ Furthermore, researchers have shown that fluorinated ethers, including bis­(2,2,2-trifluoroethyl) ether (BTFE),^13^ 1,1,2,2-tetrafluoroethyl-2,2,3,3-tetrafluoropropyl ether (TTE),^14^ 1,1,2,2-tetrafluoroethyl-2,2,2-trifluoroethyl ether (TFTFE),^15^ and 1,3-(1,1,2,2-tetrafluoroethoxy)­propane (FDE),^16^ can improve Li–S battery electrolytes.

In parallel with advances in liquid electrolyte formulation, growing attention has been directed toward solid and quasi-solid-state electrolytes to address the safety limitations of conventional liquid systems. Recent reviews have comprehensively summarized the progress of all-solid-state and quasi-solid-state electrolytes for Li–S batteries, highlighting their roles in suppressing polysulfide shuttling and lithium dendrite growth.^17^ While liquid electrolytes typically offer high ionic conductivity and excellent electrode wettability, they also pose significant safety concerns due to their flammability and the risk of leakage. On the other hand, solid-state electrolytes are inherently safer and mechanically robust, which helps suppress dendrite formation and enhances battery safety. However, their practical application is often limited by low ionic conductivity and poor interfacial contact with the electrodes, leading to high interfacial resistance and limited cycling performance. Quasi-solid-state electrolytes, particularly gel polymer electrolytes (GPEs), offer a promising middle ground by combining the favorable ion transport properties of liquid electrolytes with the mechanical stability and safety features of solids. These systems typically consist of a polymer matrix swollen with liquid or semisolid electrolyte, enabling improved interfacial contact, better mechanical integrity, and reduced volatility.^18,19^ Moreover, incorporating fluorinated ether solvents into quasi-solid matrices can enhance electrochemical stability and suppress polysulfide shuttling, making them particularly suitable for high-performance Li–S battery systems.

The development of optimized electrolyte systems remains a slow and resource-intensive process, largely due to reliance on traditional trial-and-error approaches. The conventional one-variable-at-a-time method, where only a single factor is varied while others are held constant, continues to be widely used despite its inherent limitations, including the need for a large number of experiments, limited generalizability of results, inability to capture interactions between variables, and practical constraints related to time and cost. Statistical Design of Experiments (DoE) has proven to be a valuable methodology for systematically exploring the relationships between experimental factors and responses, enabling more efficient data collection and robust analysis. In recent years, DoE has been successfully applied across various aspects of lithium-ion battery research, including degradation of cells,^20^ energy capacity optimization,^21^ electrolyte formulation,^22^ synthesis conditions,^23^ state of charge,^24^ thermal behavior,^25^ and model parametrization,^26^ demonstrating its broad utility in advancing battery technology through efficient and rigorous experimentation. Within the DoE framework, statistical models such as Gaussian process regression (GPR) are often employed to establish relationships between experimental factors and responses. These models have been applied in lithium-ion battery (LIB) studies for tasks such as *in situ* capacity estimation,^27^ cyclic capacity prediction,^28^ and state-of-health (SOH) estimation.^29^ Only a few reports using DOE in lithium sulfur systems as lithium polysulfide absorption^30^ and cathode formulation^31^ have been published to date.

Building on this foundation, this study applies a statistical DoE strategy integrated with mixture design methods to efficiently explore and optimize the electrolyte composition for quasi-solid-state Li–S batteries. Seven quasi-solid electrolyte formulations were selected using a ternary mixture design comprising different volume ratios of DOL, DME, and OTE, ensuring comprehensive coverage of the design space with minimal experimental effort. These formulations were screened to investigate the interplay between solvent composition and electrochemical performance, particularly emphasizing the role of fluorinated ethers. The addition of fluorinated ethers aims to reduce capacity fading by mitigating polysulfide dissolution, while the polymer matrix enhances mechanical stability and suppresses electrolyte leakage. Key performance indicators—including ionic conductivity, initial discharge capacity, and capacity fading—were evaluated to assess electrolyte effectiveness. A composite performance metric was developed to balance these objectives. To quantitatively capture the relationship between electrolyte composition and performance, a GPR model was trained on the experimental data. To the best of our knowledge, this work represents the first implementation of multivariable GPR within a DoE framework for Li–S batteries. The model enabled the prediction of performance across the compositional space and guided the identification of the optimal electrolyte formulation, denoted as composition, DOL:OTE:DME = 0.273:0.505:0.222, within the targeted design range. This DoE approach, coupled with GPR modeling, provides an efficient and data-informed path to optimize electrolyte composition while minimizing the number of required experiments.

## 2 Experimental Section

### 2.1 Electrolyte Preparation

To prepare the liquid electrolyte, 1 M lithium bis­(trifluoromethanesulfonyl)­imide (LiTFSI, LiC_2_F_6_NO_4_S_2_, 99.5%, MSE Supplies) was dissolved in a nonaqueous solvent mixture consisting of 1,3-dioxolane (DOL, C_3_H_6_O_2_, Sigma-Aldrich), 1,2-dimethoxyethane (DME, C_4_H_10_O_2_, Sigma-Aldrich), and 1*H*,1*H*,5*H*-octafluoropentyl-1,1,2,2-tetrafluoroethyl ether (OTE, C_7_H_4_F_12_O, Synquest Laboratories), in desired volume ratios. The mixture was stirred at 300 rpm overnight to ensure complete dissolution of the salt. Separately, a monomer solution was prepared by mixing 99 wt % polyethylene glycol dimethacrylate (PEGDMA, (C_2_H_4_O)_*n*_(C_4_H_5_O_2_)_2_, *M*_*n*_ = 550 g mol^–1^, Oakwood Chemicals) with 1 wt % azobis­(isobutyronitrile) (AIBN, C_8_H_12_N_4_, Sigma-Aldrich) as the thermal initiator. This mixture was stirred at 300 rpm for 1.5 h until a clear solution was obtained. The prepared liquid electrolyte and monomer solution were then mixed in a 95:5 volume ratio and stirred at 300 rpm for an additional 1.5 h to form a homogeneous monomer slurry. Polymerization was carried out at 50 °C for 2 h to produce the quasi-solid electrolyte. All solvents were predried using 3 Å molecular sieves before use. All procedures were performed in an argon-filled glovebox (MBraun), with moisture and oxygen levels maintained below 1 ppm.

### 2.2 Experimental Design

To investigate the effect of electrolyte composition on Li–S battery performance, a modified mixture design approach was employed based on the principles of DOE. A ternary phase diagram was constructed using three ether liquid components: DOL, OTE, and DME. Although this approach is based on a three-component mixture design, it was intentionally adapted to minimize the number of experimental runs while still achieving even and representative coverage of the targeted compositional space.

The design avoids vertex points of the ternary diagram to exclude single-solvent formulations and instead focuses on combinations that contain multiple components, ensuring a balanced evaluation of solvent interactions within the system. Single-solvent formulations were intentionally excluded, as they do not capture the combined effects between solvents and are often unsuitable for practical use due to limitations in conductivity, solubility, and/or electrochemical stability. A 1:1 volume ratio of DME and DOL is commonly used in sulfur batteries to balance their respective roles: DME improves ionic conductivity and redox kinetics at the sulfur cathode, while DOL suppresses LiPS solubility and promotes stable SEI formation at the lithium anode. A fluorinated ether (OTE) was also included to further reduce polysulfide solubility and support stable cycling performance. All selected compositions used for performance evaluation were constrained to a maximum of 50 vol % for each component. This region was chosen to promote synergistic interactions among all three components, where intermediate mixtures offer a balance between mechanical integrity, ionic conductivity, and electrochemical stability. The distribution of design points across the composition space is shown in the phase triangle, as illustrated in Figure 1 (ternary electrolyte design section). Table 1 provides the volume ratios of DOL, OTE, and DME used in each electrolyte formulation. Each quasi-solid electrolyte will hereafter be referred to using the label provided in Table 1.

**Table 1:** Volume Ratios of DOL, OTE, and DME in the Seven 1 M LiTFSI Liquid Electrolyte Compositions Used to Prepare the Quasi-Solid Electrolytes

design points	DOL (vol %)	OTE (vol %)	DME (vol %)	electrolyte label
1	0.500	0.500	0	110
2	0	0.500	0.500	011
3	0.500	0	0.500	101
4	0.500	0.250	0.250	211
5	0.250	0.500	0.250	121
6	0.250	0.250	0.500	112
7	0.333	0.333	0.333	111

**Figure 1:**
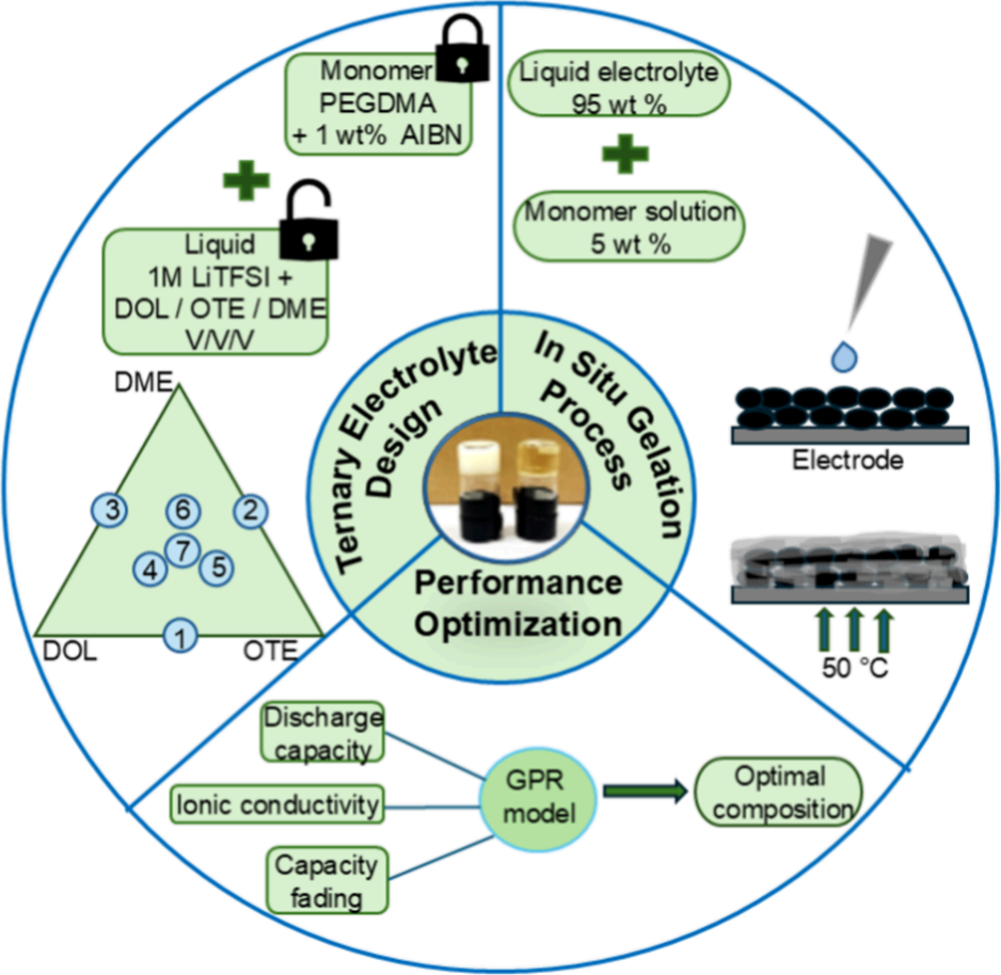
Schematic illustration of the experimental workflow to design an optimal quasi-solid-state electrolyte composition for Li–S cells. The process involves (1) ternary electrolyte formulation design based on the DOL–OTE–DME phase space; (2) *in situ* gelation via thermally initiated polymerization; and (3) performance optimization guided by a composite performance metric.

To evaluate and compare the performance of the seven electrolyte formulations, a composite performance score was calculated based on the initial discharge capacity, ionic conductivity, and capacity fading over the first 10 cycles. These early cycle parameters were selected as reliable indicators of initial electrochemical behavior and were used to train the GPR model for electrolyte composition optimization. A composite performance score was defined to balance three key factors—initial discharge capacity, lithium-ion transport kinetics, and cycling stability—to identify the electrolyte formulation with the most favorable overall performance. The score was calculated as eq 1:

$$\mathrm{Score}=\frac{\mathrm{Initial discharge capacity}\times \mathrm{Ionic conductivity}}{\mathrm{Capacity fading}}$$ 1

Here, capacity fading refers to the total loss in discharge capacity measured over the first 10 cycles, which measures the electrolyte’s stability during early cycling. The composition with the highest predicted score was identified as the optimal composition. Extended cycling and rate performance tests were carried out using the optimized electrolyte. For comparison, the baseline 101 electrolyte was also tested to validate the electrochemical performance of the Li–S cell.

### 2.3 Cathode Preparation

Sulfur powder (<99%, Sigma-Aldrich) was mixed with carbon nanotubes (CNTs, Sigma-Aldrich) (sulfur:CNTs = 3:1) in ethanol through a THINKY mixer (1800 rpm, 20 min). The obtained mixture was heated at 70 °C for 24 h to remove any trapped ethanol. The S@CNT nanocomposite was prepared following a melt-diffusion strategy. The mixture was sealed in an argon-filled Teflon reactor and heated at 155 °C for 12 h to obtain the S@CNT composite. *N*-Methyl pyrrolidone (NMP, Sigma-Aldrich) was used as the solvent to prepare the slurry. To fabricate the S@CNT electrode, S@CNT composite material, acetylene black (AB, Sigma-Aldrich), and polyvinylidene fluoride (PVDF, Sigma-Aldrich) were mixed in a weight ratio of 7:2:1. The S@CNT composite was first mixed with AB and ground for 30 min. PVDF was dissolved in NMP separately, and the ground mixture was then added and stirred vigorously for 72 h to form a viscous slurry. The slurry was coated onto a carbon-coated aluminum foil and dried in a vacuum oven for 12 h, followed by additional 12 h of drying at 60 °C in a vacuum oven. Disks with a diameter of 12.7 mm were then punched out from the dried coating. The active material (sulfur) weight in the electrode is about 0.4 mg/cm^2^.

### 2.4 Cell Assembly

CR2032-type Li–S coin cells were assembled in an Ar-filled glovebox. The PEGDMA-based polymer electrolyte was fabricated *in situ* via direct polymerization. The cells were assembled using a sulfur cathode, a polypropylene (PP, Celgard, 20 μm) separator, and a lithium chip as the anode. For cell assembly, 25 μL of the monomer slurry, as described in Section 2.1, was dropped onto the cathode. The PP separator was then placed on top of the cathode, followed by the addition of 15 μL of the monomer slurry. A lithium chip was subsequently placed on the separator. After stacking, the cells were crimped and left to rest for 24 h to ensure thorough wetting of the electrodes by the precursor solution. The cells were then placed in an oven preheated to 50 °C and maintained at that temperature for 2 h to complete polymerization. After *in situ* polymerization, the cells were cooled down to room temperature and rested for an additional 24 h before testing. All electrochemical measurements were taken at 45 °C.

### 2.5 Physical Characterization

The structural characterization of S@CNTs was performed using XRD patterns of elemental sulfur, bare CNTs, and the as-synthesized S@CNT composite. The XRD patterns were collected using a Bruker D2 PHASER diffractometer in Bragg–Brentano geometry, employing Ni-filtered Cu Kα radiation (1.5405 Å) and a LynxEye 1D detector with a step size of 0.01° 2θ. Thermogravimetric analyses (TGA) were performed using a thermal analyzer (METTLER TOLEDO) under nitrogen protection with a heating rate of 5 °C/min. The morphologies of the S@CNT composite were observed with scanning electron microscopy (SEM, Hitachi S-4700).

UV–visible absorption spectroscopy (PerkinElmer Lamda 950) was used to investigate the solubility of polysulfide species in different solutions. The selected LiPS species Li_2_S_8_ were prepared by mixing sulfur powder (Sigma-Aldrich) and Li_2_S powder (Sigma-Aldrich) with a molar ratio of 7:1 in a DME solution. The precursor solution was heated at 45 °C and stirred for 48 h. Polysulfide adsorption tests were performed by adding a 5 mM Li_2_S_8_ solution to the prepared electrolytes.

### 2.6 Electrochemical Measurements

Electrochemical impedance spectroscopy (EIS) was conducted to measure the ionic conductivities of the electrolytes. The measurements were performed using a GAMRY Interface 1010E in a frequency range of 0.1 to 2 × 10^6^ Hz, with an AC signal amplitude of 10 mV. For these tests, CR2032-type coin cells were assembled using two stainless steel (SS) electrodes in a blocking configuration (SS/E/SS). The ionic conductivity of the prepared electrolyte was calculated using eq 2.

$$\sigma =\frac{d}{R_{b}S}$$ 2

where σ is the ionic conductivity, *R*_b_ is the bulk resistance, *d* is the thickness of the quasi-solid electrolyte, and *S* is the area of the electrode.^32^ For temperature-dependent conductivity analysis, the cells were equilibrated at each temperature for 2 h prior to testing. EIS measurements were then performed sequentially at 15, 25, 35, 45, and 55 °C.

To determine the electrochemical stability window of the electrolytes vs Li/Li^+^, linear sweep voltammetry (LSV) was performed on SS|electrolyte|Li cells, scanned from 2 to 5 V at a rate of 0.1 mV s^–1^. Galvanostatic cycling tests were carried out on Li/GPE/Li symmetric cells using a NEWARE Battery Tester, with a current density of 0.1 mA cm^–2^ and a half-cycle duration of 1 h. For full-cell testing, the Li–S cells were cycled at a rate of C/10 (1 C = 1675 mAh g^–1^) in the voltage range of 1.5–2.6 V. To monitor the resistance evolution of the SEI layer during cycling, EIS measurements were conducted at three stages: before cycling, after 5 cycles, and after 10 cycles. To further evaluate the Li–S cell, rate performance tests were conducted at 0.1, 0.2, 0.3, and 0.5 C, with 10 cycles performed at each rate, followed by a return to 0.1 C for 10 cycles. These tests were carried out for both the cell with the optimized electrolyte composition and the one with the baseline electrolyte. Additionally, galvanostatic charge–discharge cycling was performed for 100 cycles at 0.3 C for both cells. All the experiments were carried out at 45 °C.

## 3 Results and Discussion

### 3.1 Structural Characterization of the S@CNT Cathode

S@CNT composite cathodes were fabricated to improve the electrical conductivity and enhance the electrochemical performance of Li–S cells. Figure S1a shows the XRD patterns of the S@CNT composite, CNTs, and elemental sulfur. All peak positions of the S@CNT composite correspond to the standard orthorhombic crystal structure of elemental sulfur, indicating that no phase transformation occurs during composite preparation, and the crystal structure of sulfur remains. The XRD pattern of CNTs exhibits two prominent peaks at 2θ = 26.05 and 44.35°, respectively. The peak of the CNT center at 26° disappeared, and the baseline of the peaks of the S@CNT composite around 26° is slightly raised. The sulfur XRD peaks were well integrated with those patterns by CNTs. The sulfur content in the S@CNT composite was determined by thermogravimetric analysis (TGA), as shown in Figure S1b. According to the TGA results, a significant weight loss was observed between 180 and 300 °C, which is attributed to the sublimation of sulfur. Based on this weight loss, the sulfur content in the S@CNT composite was calculated to be approximately 75 wt %. As shown in the SEM image in Figure S1c, the S@CNT composites retain the tubular structure of the CNTs. Numerous CNTs, each several micrometers in length, are entangled to form a net-like structure. Such a porous structure with interconnected channels may help alleviate the volume expansion of sulfur during cycling,^33^ which is expected to enhance the electrochemical performance of Li–S batteries, including cycling stability and rate capability.

### 3.2 Gelation and Structural Behavior of Electrolytes

All seven quasi-solid electrolytes were synthesized using an in situ polymerization method. The polymerization was initiated thermally by activating 1 wt % AIBN at 50 °C—within the standard 50–60 °C range used for AIBN-initiated PEGDMA cross-linking—triggering rapid chain propagation and network formation in the monomer.^34,35^ This polymer matrix serves as the structural framework of the quasi-solid electrolyte. For all seven electrolytes, the mixing ratio of liquid electrolyte to monomer solution was fixed at 95:5, and the monomer solution composition (PEGDMA with 1 wt % AIBN) was also kept constant. Only the volume ratios of DOL, OTE, and DME within the liquid electrolyte were varied to explore their compositional effects.

Figure 2a illustrates a schematic representation of the in situ polymerization process, where the monomer solution (PEGDMA + AIBN) and liquid electrolyte components are thermally cross-linked to form a quasi-solid gel matrix. Figure 2b presents the optical images of the seven different quasi-solid electrolytes after gelation. Electrolytes with higher OTE volume content remain more stable and retain their shape near the top of the vial when inverted. In contrast, the electrolyte containing only DOL and DME tends to flow downward along the vial wall, demonstrating a less solid structure. Furthermore, electrolytes with higher OTE concentration appear cloudy, while those with a lower OTE concentration remain transparent. The cloudiness observed in OTE-rich electrolytes is likely indicative of microphase separation caused by the limited compatibility of OTE with DOL and DME. This visual turbidity suggests the occurrence of polymerization-induced phase separation (PIPS). A similar phenomenon was reported by Lu et al., where fluorinated acrylate monomers promoted phase separation from the SN-LiTFSI solution during *in situ* polymerization, resulting in a bicontinuous structure that enhanced the mechanical properties of the gel polymer electrolyte.^36^

**Figure 2:**
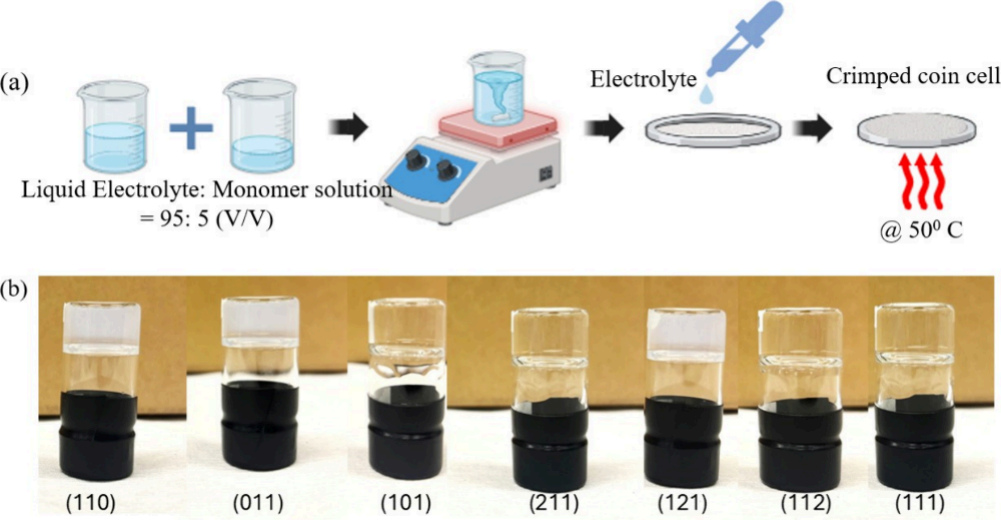
(a) Schematic illustration of the coin cell assembly process via *in situ* polymerization. (b) Optical images of the quasi-solid electrolytes after *in situ* polymerization (2 h at 50 °C) and subsequent resting at room temperature for 24 h. The vials are shown in the inverted position to observe the physical stability of the formed electrolytes.

### 3.3 EIS and Conductivity

Quasi-solid electrolytes containing different volume ratios of liquid components were characterized using impedance spectroscopy with two SS electrodes as blocking electrodes. As shown in Figure 3a, the Nyquist plots of the electrolytes exhibit two regions: a compressed semicircle in the high-frequency region and a linear region in the low-frequency range. The semicircle at high frequency corresponds to ion conduction in the bulk of the electrolyte, while the linear part at low frequency is attributed to the effect of the blocking electrodes.^37^ The ionic conductivity of the electrolytes was calculated from the impedance plots, and it was taken as the resistance values corresponding to the intercept of the linear fit of the straight line with the *x*-axis (e.g., when the imaginary part is equal to zero).^38^ The series of electrolytes exhibit ionic conductivities in the range of 0.074 to 0.756 mS/cm at 45 °C. Notably, the conductivity of the 111 electrolyte is approximately 10 times higher than that of the 110 electrolyte. Consistent with previous reports, DOL is generally associated with lithium metal passivation, while DME—owing to its relatively higher donor number and lower viscosity—facilitates enhanced ionic transport,^39,40^ which is likely the reason why electrolytes with higher DOL content exhibited lower ionic conductivity compared to those with higher amounts of DME.

**Figure 3:**
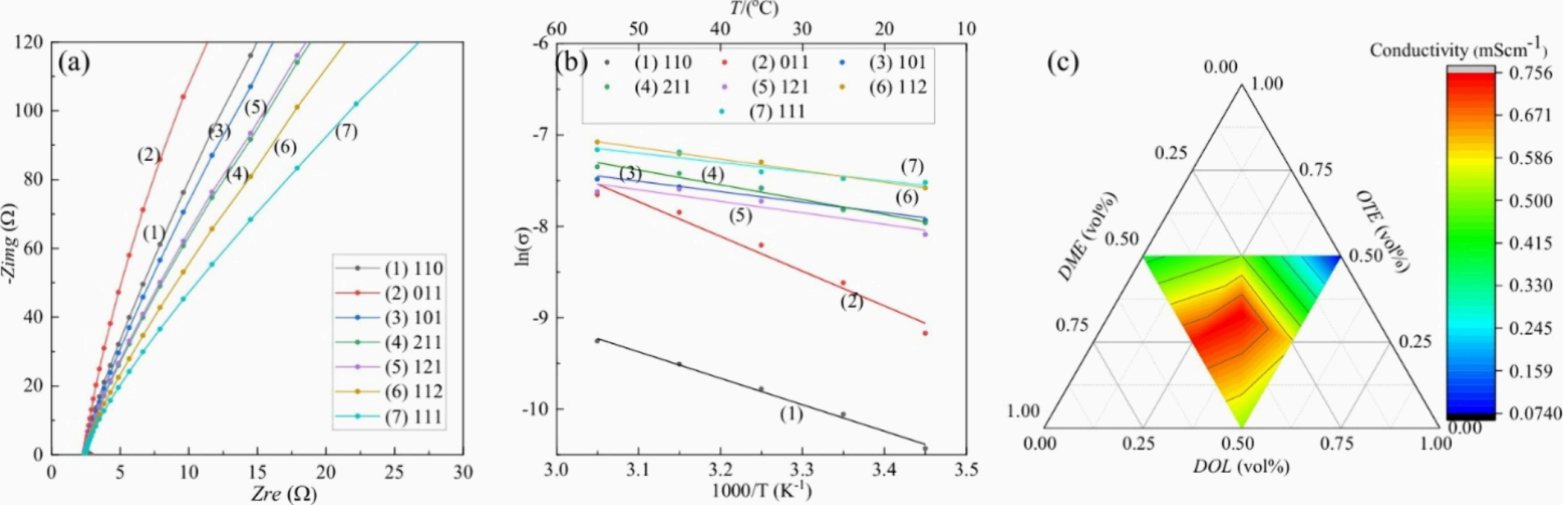
(a) EIS plots of quasi-solid electrolytes at 45 °C using the coin cell configuration SS/E/SS. (b) Temperature dependency of the ionic conductivities of electrolytes over the temperature range 15 to 55 °C. (c) Ternary plot showing the ionic conductivity distribution of quasi-solid electrolytes at 45 °C as a function of the volume ratio of DOL, OTE, and DME. Each point represents a unique electrolyte composition, with conductivity values indicated by the color scale.

The Arrhenius model explains the relationship between ionic conductivity and temperature, which can usually be expressed as follows:^41^

$$\sigma =A_{0}\exp\left(\frac{-E_{a}}{RT}\right)$$ 3

where *E*_a_ is the activation energy, *R* is the gas constant, and *A*_0_ is the pre-exponential factor. The σ values of the electrolytes obtained using Nyquist plots are used to construct ln­(σ) versus 1000/T plots, as shown in Figure 3b. The linearity of the plot indicates that the data for the set of electrolytes follow Arrhenius behavior, confirming the validity of the Arrhenius equation (eq 3). Using the value of the slope and eq 3, calculated *E*_a_ values are in the range between 0.328 and 0.087 eV. Table 2 presents the activation energies calculated from the Arrhenius plots of EIS data, along with the ionic conductivity values measured at 45 °C for each quasi-solid electrolyte composition. The 111 electrolyte exhibits the lowest activation energy (0.087 eV) and the highest ionic conductivity at 45 °C (0.756 mS cm^–1^), showing enhanced properties compared to the baseline electrolyte DOL:DME = 1:1 (101). Since lower activation energy is directly correlated with faster lithium-ion diffusion,^42^ the 111 electrolyte appears to be the most favorable in terms of ionic transport performance among the series of electrolytes.

**Table 2:** Activation Energies Calculated from the Arrhenius Plots of EIS Data and Ionic Conductivity Values at 45 °C for Each Quasi-Solid Electrolyte Composition

electrolyte label	activation energy (eV)	ionic conductivity 45 °C (mS cm^–1^)
110	0.250	0.074
011	0.328	0.391
101	0.098	0.516
211	0.140	0.598
121	0.108	0.503
112	0.109	0.741
111	0.087	0.756

### 3.4 Electrochemical Stability vs Lithium Metal

To determine the electrochemical stability window of the series of electrolytes, LSV measurements were conducted using the Li/E/SS cell configuration and the obtained results are presented in Figure 4. All seven quasi-solid electrolytes exhibit electrochemical stability beyond 3.5 V vs Li/Li^+^ at 45 °C. The electrolyte without DME remains stable up to 4.5 V, showing the highest voltage tolerance among the tested formulations. These findings are consistent with reported studies that provide clear evidence of DOL’s ability to form a stable SEI on the lithium metal surface, whereas DME is more reactive toward lithium.^43,44^ The electrolytes containing all three components—DOL, OTE, and DME—undergo side reactions at around 3.5–3.6 V vs Li/Li^+^. Since the typical charge–discharge voltage window of a Li–S battery lies between 1.5 and 3.0 V vs Li/Li, these electrolytes exhibit oxidative electrochemical compatibility within the operational voltage range of Li–S cells.

**Figure 4:**
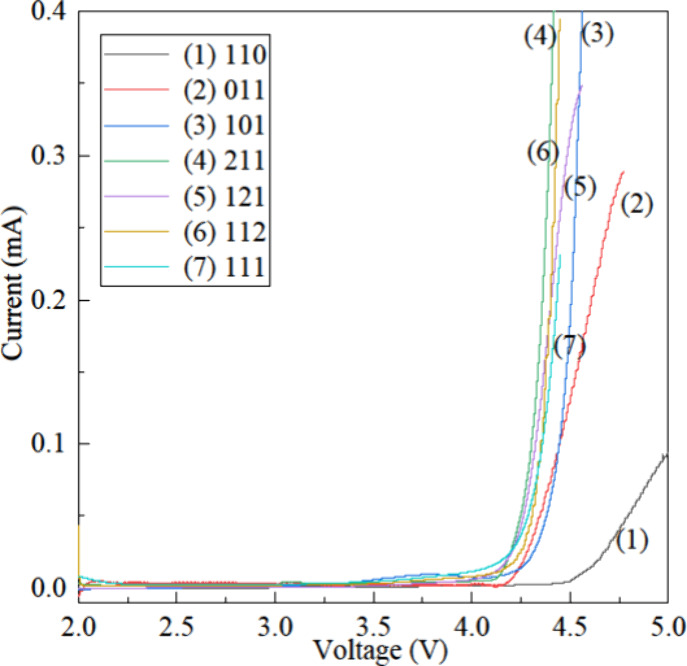
LSV curves of the seven electrolyte formulations, used to evaluate their electrochemical stability against the lithium metal anode in a Li/E/SS cell configuration.

The lithium plating and stripping behavior of all seven quasi-solid electrolytes was evaluated in symmetric lithium cells at 45 °C under a current density of 0.1 mA cm^–2^ to assess their long-term stability. As shown in Figure 5a,c, all cells demonstrate stable lithium plating and stripping behavior over an extended period of 600 h, with no significant fluctuations in overpotential observed during cycling. The voltage profiles presented in Figure 5b,d further confirm the smooth and consistent deposition and dissolution of lithium, showing no signs of short circuits, which would lead to a sudden drop in the cell voltage. All seven electrolyte formulations maintain overpotentials below 50 mV throughout the testing period, indicating efficient interfacial stability. Notably, the 101 electrolyte, which does not contain OTE, exhibits the lowest overpotential of approximately 10 mV over 600 h of continuous cycling.

**Figure 5:**
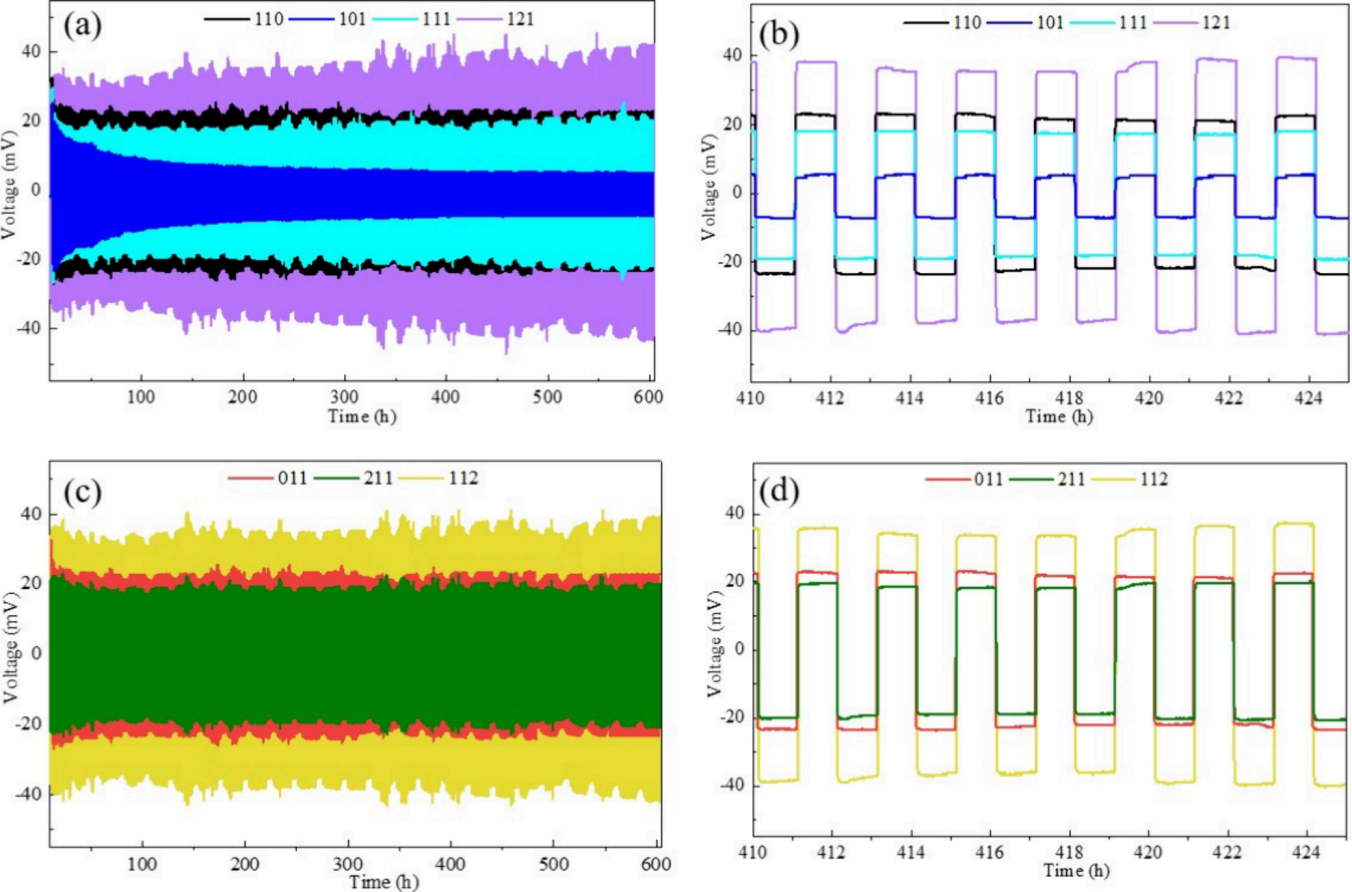
Galvanostatic cycling stability of Li∥Li symmetric cells in electrolytes at 0.1 mA cm^–2^. (a) Voltage profiles in 110, 101, 111, and 121 electrolytes over 600 h. (b) Zoomed-in view of the voltage response between 410 and 425 h. (c) Voltage profiles in 011, 211, and 112 electrolytes over 600 h. (d) Zoomed-in view of the voltage response between 410 and 425 h to highlight detailed behavior during prolonged cycling.

### 3.5 Lithium Polysulfide Diffusion

The solubility of LiPSs in the quasi-solid electrolytes was evaluated by adding Li_2_S_8_ to each electrolyte formulation. As shown in Figure S2, the polysulfides did not fully penetrate the quasi-solid electrolytes, even after 1 week. All samples exhibited a two-layer structure, with the upper layer containing the polysulfide solution and the lower layer consisting of the electrolyte. In the 110 and 011 electrolytes, a thin brown layer remained on the surface, indicating minimal polysulfide infiltration. This behavior is likely due to the higher OTE content in these formulations, which forms a more rigid gel structure that hinders polysulfide diffusion. In contrast, other electrolytes formed a thicker yellow layer on the top, suggesting their gel texture allows partial penetration of polysulfides into the electrolyte. Among these, the 211 and 121 electrolytes showed lighter-yellow coloration compared to 101, 112, and 111, suggesting lower polysulfide dissolution in 211 and 121. The darker-yellow appearance observed in 101 and 112 may be attributed to their higher DME content, which likely enhances the solubility of polysulfides.

Since the quasi-solid electrolytes did not allow full infiltration of polysulfides, UV–vis spectroscopy was performed on four selected liquid electrolyte formulations containing different combinations of DOL, OTE, and DME to evaluate the effect of electrolyte component ratio on polysulfide dissolution. As shown in Figure S3, the 101 electrolyte exhibits a brown color, indicating higher polysulfide solubility. In contrast, the other three electrolytes—110, 011, and 111—which contain OTE, appear yellow, suggesting reduced polysulfide dissolution. Among these, the 101 electrolyte shows the highest absorbance in the UV–vis spectra, consistent with its darker color. 110 appears pale yellow and shows the lowest absorbance, indicating minimal polysulfide solubility. These results from both visual observation and UV–vis analysis highlight the influence of solvent composition, particularly the introduction of fluorinated ether (OTE) on polysulfide solubility.

### 3.6 Electrochemical Performance of Li–S Cells

Figure 6 presents the charge–discharge cycling performance of Li–S cells incorporating all seven quasi-solid-state electrolytes. Ternary phase diagrams of DOL, OTE, and DME were constructed to visualize composition-dependent trends. The corresponding initial 10 charge–discharge profiles for each electrolyte formulation are provided in Figure S4. The cell using electrolyte 111 exhibits the highest first discharge capacity, while the cell using electrolyte 110 delivers the lowest discharge capacity among the tested formulations. Notably, the 111-electrolyte-based cell also shows the most significant capacity fading over 10 cycles, whereas the 110-electrolyte-based cell demonstrates the most stable cycling performance with the least fading.

**Figure 6:**
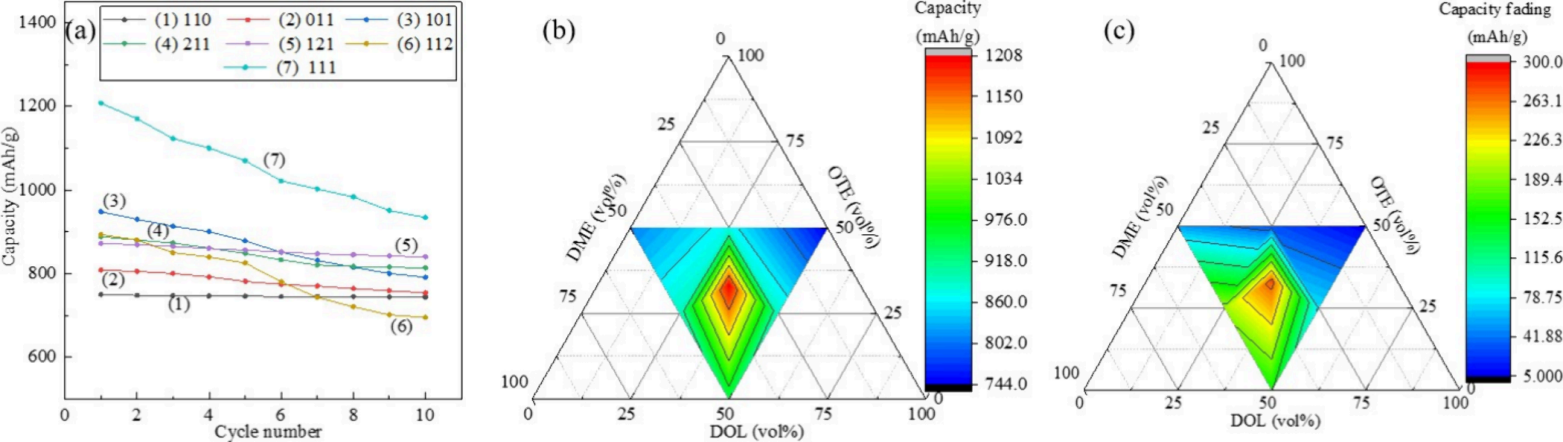
Electrochemical performance. (a) Discharge capacity for all seven quasi-solid Li–S cells over the first 10 cycles measured at a cycling rate of 0.1C. Ternary plots showing (b) first discharge capacity distribution and (c) capacity fading over the first 10 cycles of quasi-solid electrolyte Li–S cells at 45 °C as a function of the volume ratio of DOL, OTE, and DME. Each point represents a unique electrolyte composition, with capacity fading values indicated by the color scale.

These trends closely correlate with the ionic conductivity data and polysulfide dissolution tests discussed earlier. Electrolyte 111, which exhibits the highest ionic conductivity, delivers high initial capacity but suffers from rapid fading. In contrast, electrolyte 110, with the lowest ionic conductivity, maintains more stable capacity over repeated cycles. A closer look at the electrolyte compositions reveals that those with higher proportions of DME particularly 112 and 111 tend to exhibit greater capacity fading. In contrast, electrolytes with a larger content of fluorinated ether—such as 110, 121, and 011—demonstrate more stable cycling with minimal capacity loss. This observation is further supported by polysulfide dissolution experiments, which show that these fluorinated ether-rich electrolytes lead to reduced polysulfide solubility, likely contributing to the improved capacity retention.

The ternary phase diagrams plotted based on the experimental data provide a clear visual representation of the relationship between electrolyte composition and electrochemical performance. In the first discharge capacity plot, the regions with the highest capacities are shown in red and are located near the center of the diagram, where the three solvent components are present in approximately equal proportions. In contrast, the lowest capacity fading is observed in the blue-colored regions, which correspond to electrolyte compositions with higher contents of DOL and OTE. These diagrams illustrate the distribution of both first discharge capacity and capacity fading across the tested formulations, offering valuable insights into how electrolyte composition impacts the initial performance of Li–S batteries.

EIS was conducted for all seven quasi-solid-state electrolytes at three stages: before cycling, after 5 cycles, and after 10 cycles. The corresponding Nyquist plots for 110, 101, and 111 are presented in Figure 7a. The spectra were fitted using an equivalent circuit model (Figure S5) to extract the values of bulk resistance (*R*_b_), SEI film resistance (*R*_SEI_), and charge-transfer resistance (*R*_ct_), as listed in Table S1. The ternary phase diagrams illustrating *R*_SEI_ and total resistance after 10 cycles are shown in Figure 7b and Figure 7c, respectively. The Nyquist plots consist of two semicircles in the high- to medium-frequency region and an inclined line in the low-frequency region. The first and second semicircles correspond to *R*_SEI_ and *R*_ct_, respectively, while the inclined line in the low-frequency region is associated with Warburg impedance (*W*), which reflects Li-ion diffusion.^45^ Among the tested formulations, the 110 electrolyte exhibited a relatively stable SEI resistance throughout cycling. However, it showed a large overall impedance, with an incomplete second semicircle and no Warburg tail in the Nyquist plot, indicating sluggish ion transport and suppressed diffusion processes. This high interfacial resistance corresponds to the lowest specific capacity observed among all electrolytes. Interestingly, despite its large impedance, the 110 electrolyte showed minimal capacity decay over 10 cycles. This stability is likely due to the formation of a highly resistive yet robust SEI layer that effectively suppresses parasitic reactions and limits polysulfide dissolution, as supported by UV–visible spectroscopy.^46^

**Figure 7:**
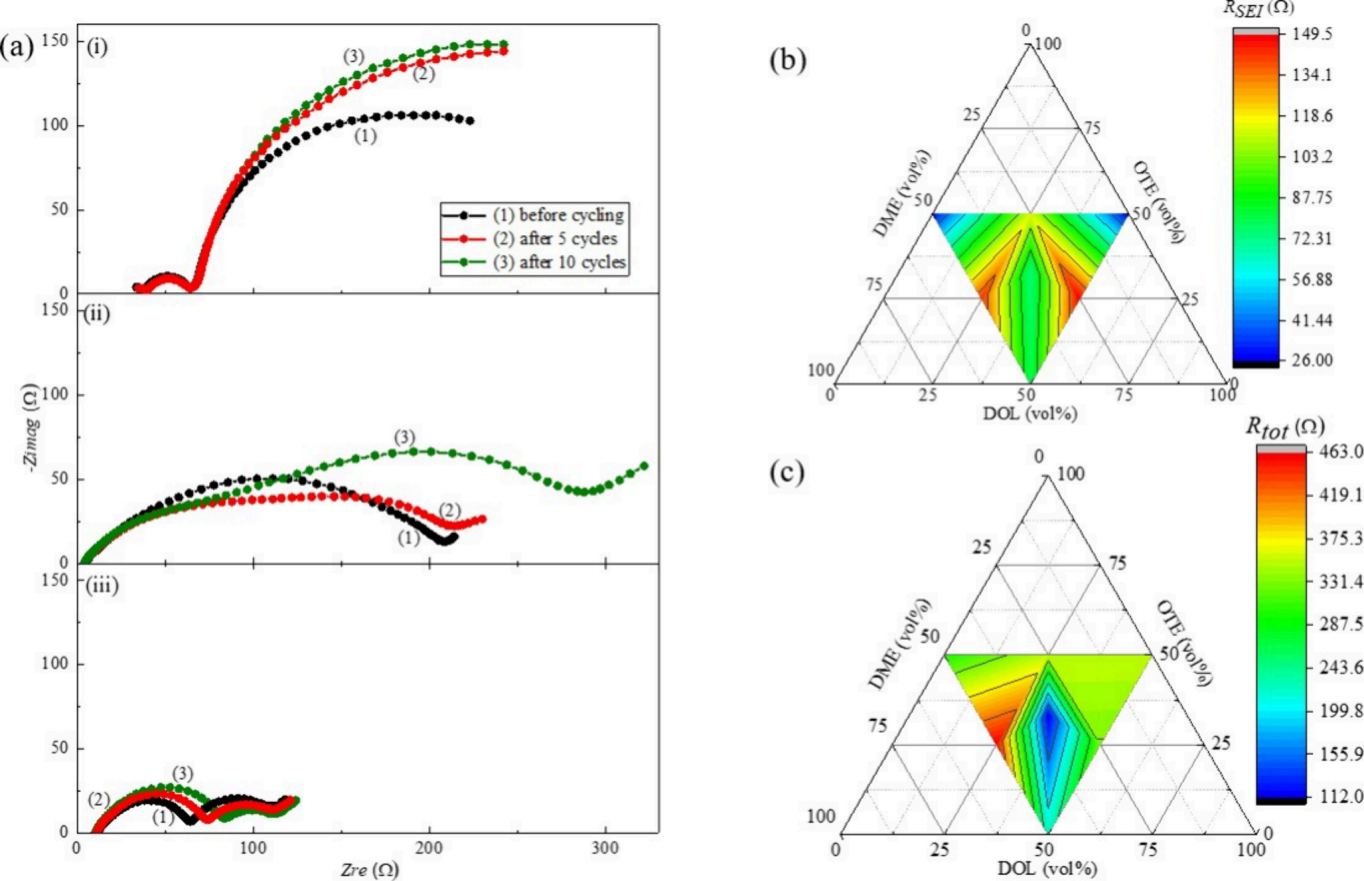
(a) Nyquist plots of Li–S cells with electrolytes (i) 110, (ii) 101, and (iii) 111, measured before cycling (black), after 5 cycles (red), and after 10 cycles (green). Ternary phase diagram showing the (b) SEI resistance (*R*_SEI_) and (c) total resistance (*R*_tot_) after 10 cycles for all seven quasi-solid-state electrolyte compositions. Both diagrams illustrate the composition-dependent trends in electrochemical resistance of Li–S cells, based on Nyquist plots obtained from EIS analysis.

In contrast, the 112 electrolyte—containing the highest proportion of DME—initially exhibited a lower total resistance than 110 but showed a substantial increase over 10 cycles, consistent with its more pronounced capacity fading. Electrolytes 121 and 112 both exhibited significant increases in SEI resistance with cycling. Notably, 112 experienced the largest increase in *R*_ct_ by the 10th cycle, suggesting deteriorating charge-transfer kinetics and more severe interfacial degradation.^47^ These findings underscore the significant impact of solvent ratios on the interfacial stability and charge-transfer behavior of quasi-solid-state electrolytes, emphasizing the need for careful electrolyte formulation to minimize resistance buildup and performance degradation over prolonged cycling.

To leverage the experimental results more effectively, a statistical modeling approach was applied to analyze performance variations and optimize electrolyte formulations within the DOL, OTE, and DME ternary composition space. A composite performance score was defined to balance three key factors—initial discharge capacity, ionic conductivity, and cycling stability—in order to identify the formulation with the most favorable overall performance. The composite scores, calculated using eq 1, are shown in Table S2 and were used to identify the optimal electrolyte composition. The GPR model revealed performance distribution patterns across the ternary space. Among the seven experimental data points, the highest observed composite score was 13.706, corresponding to the composition DOL:OTE:DME = 0.250:0.500:0.250. The ternary contour map of the performance score (Figure 8a) illustrates the performance scores of the series of electrolyte formulations. After training, the GPR model predicted an optimal composition of DOL:OTE:DME = 0.273:0.505:0.222 with a slightly improved score of 13.780, indicating potential enhancement beyond the experimentally tested points. As shown in Figure 8a, region 24 with a higher volume ratio of OTE exhibits a higher performance score, as indicated by the red color.

**Figure 8:**
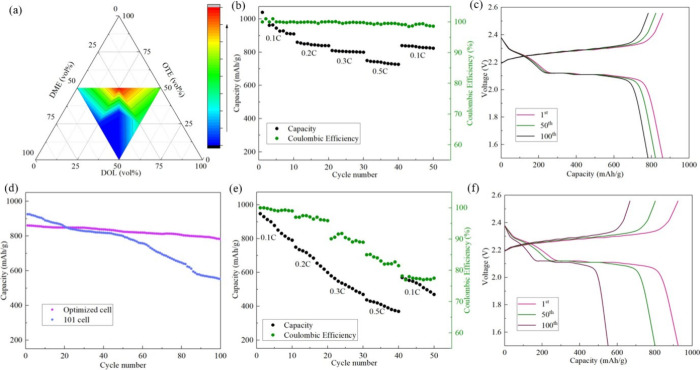
(a) Ternary phase diagram showing the composite performance scores across the electrolyte compositional space defined by DOL, OTE, and DME volume ratios. Color indicates the performance score, with higher scores shown in red and lower scores in blue. The diagram highlights the optimal region within the ternary system based on initial discharge capacity, ionic conductivity, and capacity fading. (b) Rate performance of the optimized quasi-solid-state Li–S cell measured at different current rates. The cell was cycled sequentially at 0.1, 0.2, 0.3, and 0.5 C for 10 cycles each, followed by a return to 0.1 C for 10 cycles. (c) Charge–discharge profiles of the optimal Li–S cell at 0.3 C for the 1st, 50th, and 100th cycles. (d) Discharge capacity as a function of cycle number over 100 cycles at 0.3 C for quasi-solid-state Li–S cells with optimized and 101 electrolytes. (e) Rate performance of the quasi-solid-state Li–S cell with the 101 electrolyte under the same testing protocol described in (b). (f) Charge–discharge profiles of the Li–S cell with the 101 electrolyte at 0.3 C for the 1st, 50th, and 100th cycles, corresponding to the conditions in (c).

To validate the predicted optimal formulation, a Li–S cell was assembled using the optimized electrolyte composition, following the same procedure described previously. Galvanostatic charge–discharge tests were performed at various C-rates to assess the electrochemical performance under different operating conditions. As shown in Figure 8b, the cell containing the optimized electrolyte delivered a high initial discharge capacity of 1040 mAh g^–1^ at 0.1 C and maintained a stable capacity across a range of current densities. Specifically, the cell was sequentially cycled at 0.1, 0.2, and 0.3 C for 10 cycles each to evaluate its rate capability. Following these tests, the cell was further cycled at 0.5 C, where it still maintained a discharge capacity above 700 mAh g^–1^, demonstrating strong rate performance.

After the high-rate cycling, the current was returned to 0.1 C to assess the capacity recovery. The cell exhibited a discharge capacity above 800 mAh g^–1^, indicating good reversibility and structural integrity of the electrode–electrolyte interface. The initial CE was 100%, and throughout the cycling tests, the CE remained consistently high, exceeding 97%, which reflects stable charge–discharge processes and minimal side reactions. To further investigate the long-term cycling stability of the optimized system, the cell was continuously cycled at 0.3C. As presented in Figure 8c, the Li–S cell with the optimized electrolyte exhibited an initial discharge capacity of 861 mAh g^–1^ and demonstrated excellent cycling stability, with only 9.2% capacity loss over 100 cycles at 0.3C. This result confirms the stable behavior of the optimized electrolyte over extended operation.

For comparison, a rate performance test was conducted on a Li–S cell using the baseline electrolyte formulation of DOL:DME = 1:1 (v/v), referred to as the 101 cell. The corresponding rate performance tests and charge–discharge profiles at 0.3 C are presented in Figure 8e and Figure 8f, respectively. At 0.1 C, the 101 cell exhibited a high initial discharge capacity of 948 mAh g^–1^, which decreased to 791 mAh g^–1^ after 10 cycles, indicating noticeable capacity fading even at low current. As the current density increased to 0.5 C, the discharge capacity dropped significantly to around 400 mAh g^–1^, highlighting rapid capacity loss under higher rates. Although the 101 cell initially showed a high CE of 100%, the CE decreased to approximately 77% during the rate performance tests. At 0.3 C, the 101 cell delivered an initial capacity of 926 mAh g^–1^, which is slightly higher than that of the optimized formulation. However, as shown in Figure 8d, over 100 cycles at 0.3 C, it experienced a substantial capacity loss of 40.4%, indicating poor long-term cycling stability. These results demonstrate that although the 101 cell can achieve a high initial capacity, its electrochemical performance rapidly deteriorates under prolonged cycling and varying current rates.

Cyclic voltammetry (CV) measurements were conducted to further compare the reaction kinetics of the optimized and baseline electrolytes at a scan rate of 0.1 mV s^–1^, and the results are presented in Figure S6. Both cells exhibit the typical characteristics of multistep electrochemical reactions between sulfur and lithium ions. The optimized cell displays nearly identical CV curves starting from the second cycle. The strong overlap of the subsequent oxidation and reduction peaks suggests that the optimized cell possesses high electrochemical stability and reversibility compared to the baseline cell. Another noticeable difference in the CV profiles is the lower intensity of the reduction and oxidation peaks in the optimized cell compared to the reference electrolyte. This indicates slower reaction kinetics in the optimized cell, most likely due to the lower concentration of polysulfides.^48^

Based on these comprehensive results, it can be concluded that the optimized electrolyte formulation provides a well-balanced performance, offering high discharge capacity along with excellent cycling stability and rate capability. In this study, a fluorinated ether was incorporated to suppress the polysulfide shuttle effect. However, when formulating an electrolyte by combining it with other solvents, it is crucial to determine the correct mixing ratios of each component in order to achieve optimal electrochemical performance. The findings highlight the effectiveness of the GPR-based optimization strategy in identifying a high-performing electrolyte composition within the DOL–OTE–DME ternary system. This approach enabled the development of a quasi-solid-state Li–S battery with significantly enhanced performance, achieved through a minimal number of experimental trials over a targeted compositional space.

A comparison with recently reported (post-2020) quasi-solid and gel electrolyte Li–S batteries (summarized in Table S3) shows that the optimized electrolyte developed in this work exhibits competitive cycling stability among analogous quasi-solid-state systems. This comparison highlights that the present electrolyte performance falls within the upper range of reported quasi-solid-state Li–S electrolytes under comparable testing conditions.

## Conclusions

This study introduces a strategically designed experimental and modeling framework for optimizing quasi-solid-state electrolytes. Experimental results and literature reports indicate that DME-rich compositions form overly soft gel-like structures, leading to accelerated capacity fading, whereas high proportions of DOL or fluorinated ether limit Li salt solubility and hinder ionic transport. By avoiding single-component dominant regions near the triangle’s vertices, the study prioritized multicomponent formulations and minimized the number of experimental trials ensuring a balanced evaluation of solvent interactions within the system while maintaining robust quasi-solid-state behavior and improved battery performance.

Among these, the formulation with DOL:OTE:DME = 0.250:0.500:0.250 delivered the highest performance metric score, leading to the prediction and subsequent validation of an optimized electrolyte blend (DOL:OTE:DME = 0.273:0.505:0.222). This composition achieved an initial discharge capacity of 1040 mAh g^–1^ and retained 782 mAh g^–1^ after 100 cycles at 0.3 C, corresponding to a low capacity loss of 9.2%, while maintaining consistent performance across multiple C-rates. Compared to recently reported quasi-solid-state Li–S batteries, the optimized electrolyte exhibited improved stability. Screened compositions were limited to 50 vol % fluorinated ether to balance its diluent role with salt solubility. The optimal formulation, slightly exceeding this threshold and located just beyond the sampled region, was model-selected and maintains stable electrochemical performance. This outcome reflects the strength of the GPR model, which was designed not only to interpolate within the sampled space but also to predict high-performing formulations in adjacent regions.

Beyond achieving these performance parameters, the study demonstrates the power of a flexible modeling framework. A composite performance metric reflects specific priorities—ionic conductivity, initial capacity, and cycling stability were used in the GPR model to identify the optimal electrolyte composition that enables effective charge–discharge performance of the Li–S cell. However, this metric can be reweighted to prioritize other considerations, such as improved safety, enhanced electrochemical stability, or optimization for specific electrode formulations. By coupling experimental precision with predictive modeling, the approach balances the interplay between precision and efficiency, accelerating the development of high-performance electrolytes for lithium–sulfur batteries. Moreover, the generalizability of the framework makes it well-suited for navigating complex multicomponent systems where synergistic interactions govern performance, expanding its relevance beyond Li–S chemistries to other emerging energy storage platforms.

## Supplementary Material



## Notes

XRD, TGA, and SEM characterization of S@CNT composites, lithium polysulfide diffusion experiments, UV–visible spectra, initial charge–discharge cycling performance for seven quasi-solid-state electrolytes, equivalent circuit model for EIS fitting, EIS parameters before and after cycling, composite performance scores for GPR modeling, CV plots of optimized and baseline cells, and comparison of recently reported quasi-solid and gel electrolyte Li–S batteries (PDF)
