# Supplementary material for: Optimization of Fluorinated Ether-Based Quasi-Solid Electrolyte Systems for Lithium–Sulfur Batteries
Source: ACS Appl Energy Mater. 2026 Mar 11;9(6):3472–83. doi: 10.1021/acsaem.6c00080 (PMC13014348; doi:10.1021/acsaem.6c00080)
Supplement: Supplementary file 1 [file ae6c00080_si_001.pdf]

Supporting Information

Optimization of Fluorinated Ether-Based Quasi-Solid Electrolyte Systems for Lithium-Sulfur Batteries

Ishani Senevirathna[a], Changlong Chen[a,b], Junquan Ou[b], Vignyatha Tatagari[b], Leon Shaw[b], Carlo U. Segre[a,b]*

[a]Department of Physics, Illinois Institute of Technology, Chicago, IL 60616, USA

[b]Department of Mechanical, Materials, and Aerospace Engineering, Illinois Institute of Technology, Chicago, IL 60616, USA

*Email: hsenevirathna@hawk.illinoistech.edu

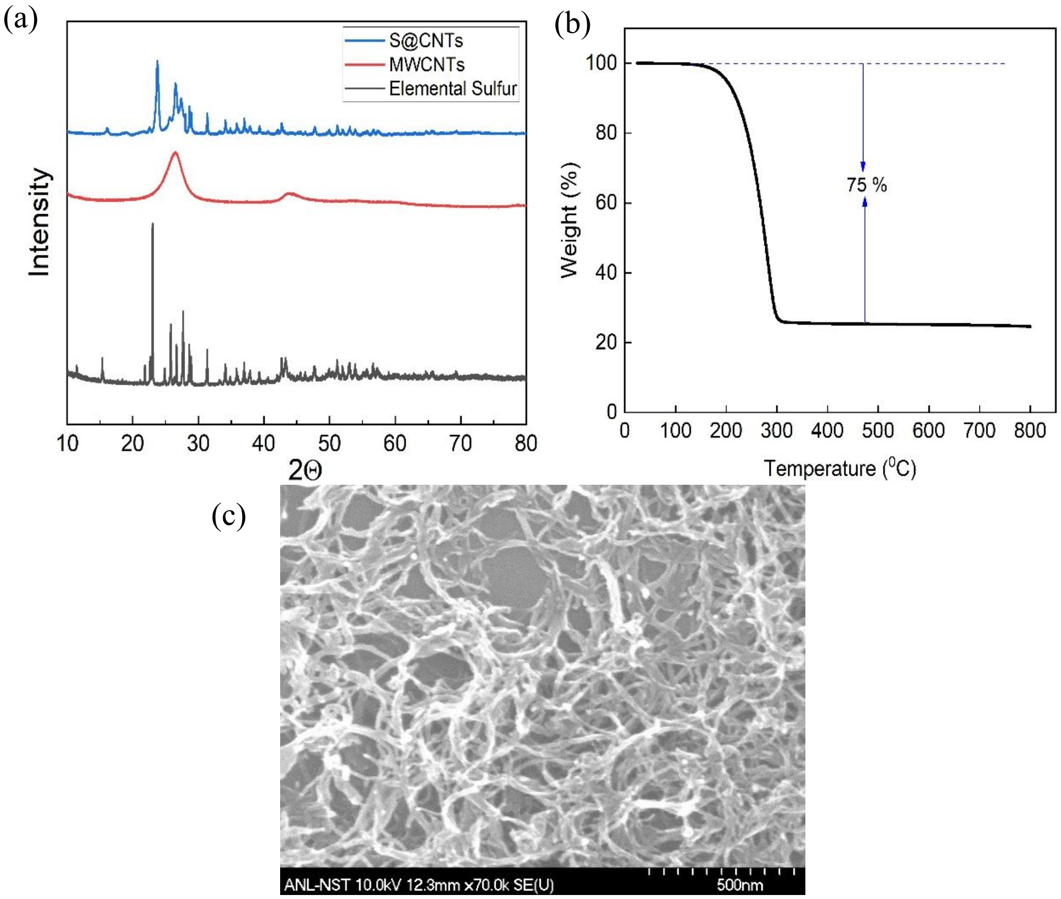

Figure S1. a) XRD patterns of S@CNTs composite (blue), pure MWCNTs (red) and elemental sulfur (black). b) TGA curves of S@CNTs composites. (c) SEM image of S@CNTs composite.

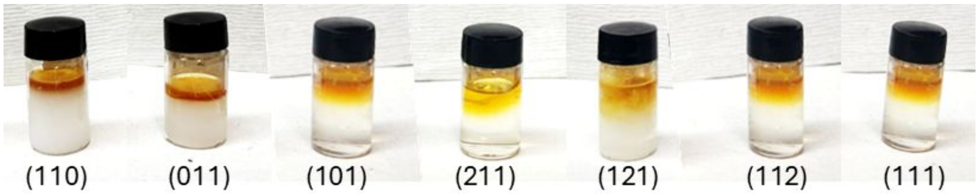

Figure S2. Lithium polysulfide diffusion experiments: the upper layer is lithium polysulfide solution, and the lower layer is quasi-solid electrolyte. Samples were kept at 45%˚C, and images were taken after one week of period.

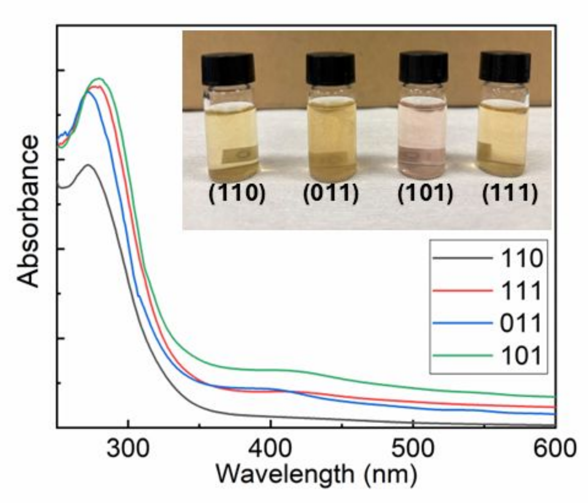

Figure S3. UV–visible spectra of four selected liquid electrolyte formulations containing different combinations of DOL, OTE, and DME after the addition of Li_2S_8, used to evaluate the effect of electrolyte component ratio on polysulfide dissolution. The inset shows optical images of the four liquid electrolyte solutions, highlighting differences in color corresponding to varying levels of polysulfide solubility.

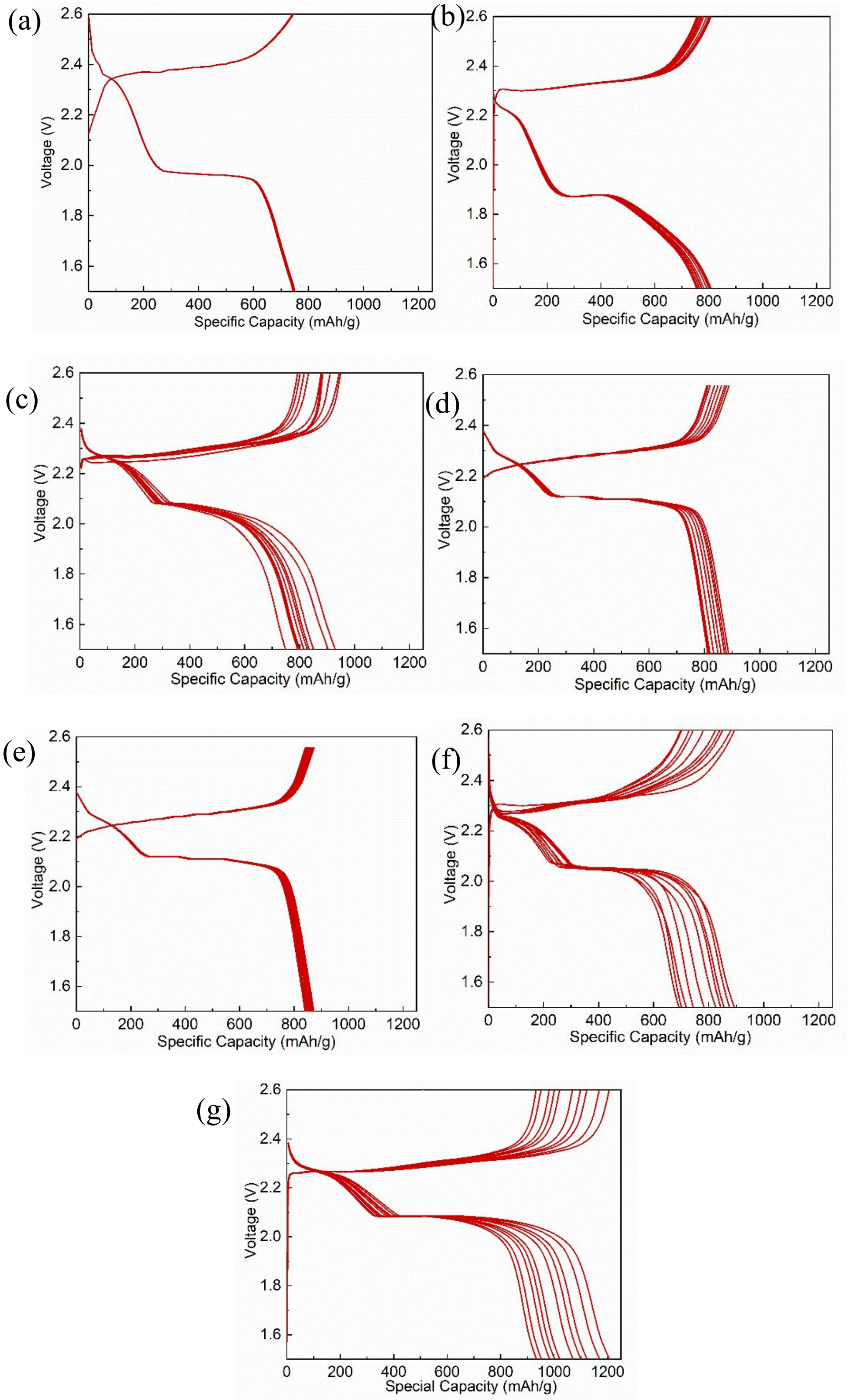
(a)
(b)
(c)
(d)
(e)
(f)
(g)
Voltage (V)
Specific Capacity (mAh/g)
Special Capacity (mAh/g)

Figure S4. Initial 10 charge–discharge cycles of Li–S cells using a) 110, b) 011, c) 101, d) 211, e) 121, f) 112, g) 111 quasi-solid-state electrolytes measured at a cycling rate of 0.1C, used to determine the optimal electrolyte composition through GPR modelling.

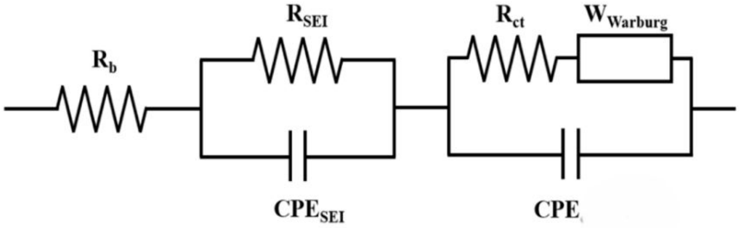

Figure S5. Equivalent circuit model used to fit the EIS plots before cycling after 5 cycles and after 10 cycles for series of electrolyte [1].

Table S1. EIS parameters for seven quasi-solid electrolyte formulations before cycling, after 5 cycles, and after 10 cycles. Parameters include bulk resistance (R_b), solid electrolyte interphase resistance (R_{SEI}), charge transfer resistance (R_{ct}) and total resistance (R_{tot}) obtained from equivalent circuit fitting.

Electrolyte label	Cycle step	R_b (Ω)	R_{SEI} (Ω)	R_{ct} (Ω)	R_{tot} (Ω)
	0	36.7	26.5	206.2	269.4
110	5	39.4	26.4	283.1	348.9
	10	39.6	26.2	286.9	352.7
	0	14.1	22.9	202.0	239.0
011	5	19.7	24.6	221.5	265.8
	10	22.2	28.3	247.2	297.7

	0	4.7	109.2	113.0	226.9
101	5	4.2	78.7	121.0	203.9
	10	4.1	79.1	140.1	223.3
	0	12.1	109.9	40.0	162.0
211	5	11.2	108.8	120.1	240.1
	10	10.8	149.2	169.8	329.8
	0	7.2	53.8	34.0	95.0
121	5	7.2	110.8	232.2	350.2
	10	7.2	113.3	232.9	353.4
	0	7.6	110.4	64.0	182.0
112	5	7.9	106.1	134.0	248.0
	10	7.7	142.3	312.1	462.1
	0	10.2	55.0	55.2	120.4
111	5	10.8	66.5	33.5	110.8
	10	11.3	73.2	27.6	112.1

Table S2. Composite performance scores used for GPR modelling. The scores were calculated using Equation 1 based on the initial experimental dataset and were employed to identify optimal electrolyte compositions within the DOL–OTE–DME ternary solvent system. The composite performance score of the GPR-predicted optimal composition is also included for comparison.

Electrolyte label	Performance score

110	9.250
011	5.850
101	3.115
211	7.080
121	13.706
112	3.342
111	3.342
Optimized Electrolyte	13.780

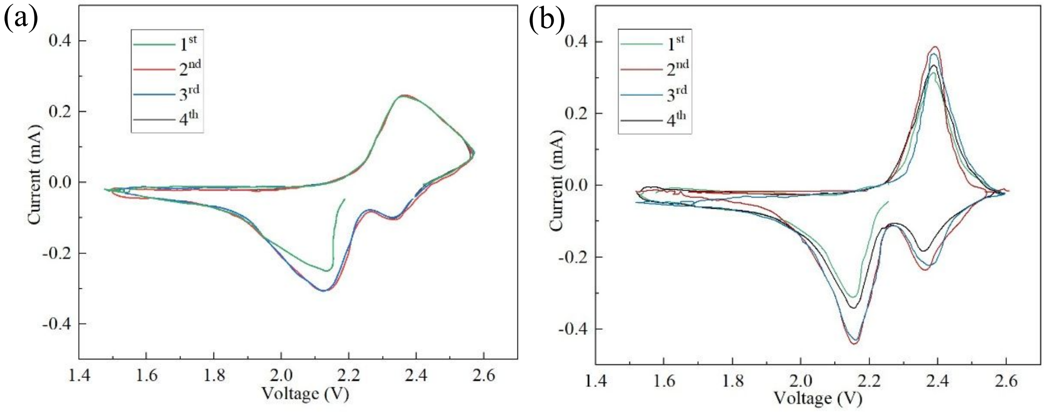

Figure S6. CV plots for optimized and baseline quasi-solid-state Li–S cell recorded at a scan rate of 0.1 mV/s.

Table S3. Comparison of representative fluorinated quasi-solid and gel electrolyte Li–S batteries

Electrolyte type	Electrolyte system	Rate	Initial Capacity (mAh/g)	Number of cycles	Capacity retention (%)	Ref.
Gel polymer	PVDF-HFP/PETT/ PETEA-DOL/DME-LiTFSI	0.2C	--	150	87.6	2
Quasi solid	PEGDE/PEI/FEC-DOL/DME-LiTFSI	0.5C	--	180	79.1	3

Gel polymer	PVDF-HFP/PVA-SiO_2	0.1C	1439	--	--	4
Gel polymer	Al_2O_3-DOL/DME-LiTFSI	0.1C	1217	--	--	5
Gel polymer	PVDF/organo-polysulfides-DOL/DME-LiTFSI/$LiNO_3$	0.5C	843	300	57.4	6
Quasi-solid	PEGDA–LLZTO-DOL/DME-LiTFSI/$LiNO_3$	0.1C	1201	100	70.2	7
Gel polymer	PVDF-HFP–Al_2O_3-DOL/DME-LiTFSI/$LiNO_3$	0.1C	1233	150	68.2	8
Gel polymer	PVDF-HFP/PETT/DA-DOL/DME-LiTFSI/$LiNO_3$	1C	630	300	88.6	9
Gel	Zein/Melamine/PAN-DOL/DME-LiTFSI	1A/g	630	400	--	10
Quasi-solid	PVDF-HFP/PSD-DME/DOL-LiTFSI	0.1C	1236	200	63.1	11
Gel polymer	PVDF-HFP-DOL/DME-LiTFSI/$LiNO_3$	0.5C	1000	105	81.5	12
Quasi solid	PEGDMA-DOL/OTE/DME-LiTFSI	0.3C	861	100	90.8	This work

"--" in the table means that this parameter is not mentioned in the paper

REFERENCES

(1) Choi, W.; Shin, H.-C.; Kim, J. M.; Choi, J.-Y.; Yoon, W.-S. Modeling and Applications of Electrochemical Impedance Spectroscopy (EIS) for Lithium-Ion Batteries. *J. Electrochem. Sci. Technol* 2020, *11* (1), 1–13. DOI: 10.33961/jecst.2019.00528

(2) Li, R.; Chen, Q.; Jian, J.; Hou, Y.; Liu, Y.; Liu, J.; Xie, H.; Zhu, J. A 3D Network-Structured Gel Polymer Electrolyte with Soluble Starch for Enhanced Quasi-Solid-State Lithium-Sulfur Batteries. *Journal of Power Sources* **2024**, *624*, 235521. DOI: 10.1016/j.jpowsour.2024.235521

(3) Zhang, T.; Zhang, J.; Yang, S.; Li, Y.; Dong, R.; Yuan, J.; Liu, Y.; Wu, Z.; Song, Y.; Zhong, Y.; Xiang, W.; Chen, Y.; Zhong, B.; Guo, X. Facile In Situ Chemical Cross-Linking Gel Polymer Electrolyte, Which Confines the Shuttle Effect with High Ionic Conductivity and Li-Ion Transference Number for Quasi-Solid-State Lithium–Sulfur Battery. *ACS Appl. Mater. Interfaces* **2021**, *13* (37), 44497–44508. DOI: 10.1021/acsami.1c16148

(4) Hu, X.; Silva, S. R. P.; Zhang, P.; Liu, K.; Zhang, S.; Shao, G. A Multifunctional Fire-Retardant Gel Electrolyte to Enable Li-S Batteries with Higher Li-Ion Conductivity and Effectively Inhibited Shuttling of Polysulfides. *Chemical Engineering Journal* **2023**, *467*, 143378. DOI: 10.1016/j.cej.2023.143378

(5) Wang, H.-M.; Fu, E.-D.; Li, G.-R.; Liu, S.; Gao, X.-P. An in Situ Fabricated Multifunctional Gel Electrolyte for Lithium–Sulfur Batteries. *Journal of Power Sources* **2023**, *581*, 233461. DOI: 10.1016/j.jpowsour.2023.233461

(6) Shen, Y.-Q.; Zeng, F.-L.; Zhou, X.-Y.; Wang, A.; Wang, W.; Yuan, N.-Y.; Ding, J.-N. A Novel Permselective Organo-Polysulfides/PVDF Gel Polymer Electrolyte Enables Stable Lithium Anode for Lithium–Sulfur Batteries. *Journal of Energy Chemistry* **2020**, *48*, 267–276. DOI: 10.1016/j.jechem.2020.01.016

(7) Shao, D.; Yang, L.; Luo, K.; Chen, M.; Zeng, P.; Liu, H.; Liu, L.; Chang, B.; Luo, Z.; Wang, X. Preparation and Performances of the Modified Gel Composite Electrolyte for Application of

Quasi-Solid-State Lithium Sulfur Battery. *Chemical Engineering Journal* **2020**, *389*, 124300. DOI: 10.1016/j.cej.2020.124300

(8) Wang, H.-M.; Wang, Z.-Y.; Zhou, C.; Li, G.-R.; Liu, S.; Gao, X.-P. A Gel Polymer Electrolyte with Al2O3 Nanofibers Skeleton for Lithium—Sulfur Batteries. *Sci. China Mater.* **2023**, *66* (3), 913–922. DOI: 10.1007/s40843-022-2252-1

(9) Hao, X.; Wenren, H.; Wang, X.; Xia, X.; Tu, J. A Gel Polymer Electrolyte Based on PVDF-HFP Modified Double Polymer Matrices via Ultraviolet Polymerization for Lithium-Sulfur Batteries. *Journal of Colloid and Interface Science* **2020**, *558*, 145–154. DOI: 10.1016/j.jcis.2019.09.116

(10) Ding, C.; Huang, L.; Guo, Y.; Lan, J.; Yu, Y.; Fu, X.; Zhong, W.-H.; Yang, X. An Ultra-Durable Gel Electrolyte Stabilizing Ion Deposition and Trapping Polysulfides for Lithium-Sulfur Batteries. *Energy Storage Materials* **2020**, *27*, 25–34. DOI: 10.1016/j.ensm.2020.01.007

(11) Jiang, J.-H.; Wang, A.-B.; Wang, W.-K.; Jin, Z.-Q.; Fan, L.-Z. P(VDF-HFP)-Poly(Sulfur-1,3-Diisopropenylbenzene) Functional Polymer Electrolyte for Lithium–Sulfur Batteries. *Journal of Energy Chemistry* **2020**, *46*, 114–122. DOI: 10.1016/j.jechem.2019.10.009

(12) Yu, J.; Liu, S.; Duan, G.; Fang, H.; Hou, H. Dense and Thin Coating of Gel Polymer Electrolyte on Sulfur Cathode toward High Performance Li-Sulfur Battery. *Composites Communications* **2020**, *19*, 239–245. DOI: 10.1016/j.coco.2020.04.015
